# Antidiabetic Effect of Brain-Derived Neurotrophic Factor and Its Association with Inflammation in Type 2 Diabetes Mellitus

**DOI:** 10.1155/2017/2823671

**Published:** 2017-09-14

**Authors:** Ceren Eyileten, Agnieszka Kaplon-Cieslicka, Dagmara Mirowska-Guzel, Lukasz Malek, Marek Postula

**Affiliations:** ^1^Department of Experimental and Clinical Pharmacology, Medical University of Warsaw, Center for Preclinical Research and Technology CEPT, Warsaw, Poland; ^2^Department of Cardiology, Medical University of Warsaw, Warsaw, Poland; ^3^Faculty of Rehabilitation, University of Physical Education, Warsaw, Poland

## Abstract

Brain-derived neurotrophic factor (BDNF) is a neurotrophin, which plays an important role in the central nervous system, and systemic or peripheral inflammatory conditions, such as acute coronary syndrome and type 2 diabetes mellitus (T2DM). BDNF is also expressed in several nonneuronal tissues, and platelets are the major source of peripheral BDNF. Here, we reviewed the potential role of BDNF in platelet reactivity in T2DM and its association with selected inflammatory and platelet activation mediators. Besides that, we focused on adipocytokines such as leptin, resistin, and adiponectin which are considered to take part in inflammation and both lipid and glucose metabolism in diabetic patients as previous studies showed the relation between adipocytokines and BDNF. We also reviewed the evidences of the antidiabetic effect of BDNF and the association with circulating inflammatory cytokines in T2DM.

## 1. Introduction

Type 2 diabetes mellitus (T2DM) is a complex condition and serious health problem worldwide. In net terms, T2DM is a group of metabolic diseases characterized by chronic hyperglycemia followed by the abnormal secretion and actions of insulin. Genetic and environmental factors are thought to be responsible for the development of T2DM [1]. Besides these, it has been noticed that T2DM is associated with inflammation [2]. Brain-derived neurotrophic factor (BDNF) is a neurotrophin (NT) that plays an important role in maturation, synaptic connection, neuronal repair, and plasticity of the central nervous system (CNS), also it has an influence on the pathology and the treatment of neurological diseases [3–7]. Besides the fundamental impact on the nervous system, several reports documented an association between plasma BDNF and systemic or peripheral inflammatory conditions, such as acute coronary syndrome and T2DM [8, 9]. BDNF has received attention, regarding a possible role in protection against the progression of T2DM [10, 11]. Some, studies suggest that BDNF may be a future target for developing new antidiabetic therapies. Herein, we review the relation between inflammatory regulation of T2DM and BDNF as a potential role in the treatment of T2DM. We also discuss some problems associated with peripheral, intrathecal, and intraventricular administration of BDNF that may greatly affect further studies and clinical administration of BDNF.

## 2. BDNF and Its Receptors

Along with the nerve growth factor (NFG) and the NT-3 and 4/5, BDNF is a member of the NT family that plays an important role in the development of the nervous system [12, 13]. It regulates the synaptic activity, neurotransmission, neuronal repair, and plasticity in many groups of mature neurons both in peripheral nervous system and CNS [14–16]. The cell surface receptors of BDNF such as p75 NT receptor (p75^NTR^), which is a member of the tumor necrosis factor receptor superfamily, and the tyrosine kinase receptor B (TrkB), a member of tropomyosine-related kinase family, mediate opposite functions on neurons [17–19]. Pro-BDNF has a high affinity to p75^NTR^, and it stimulates neuronal apoptosis [20, 21]. By contrast, mature BDNF is considered as the biologically active form, which has a high affinity to the TrkB receptor. It promotes development and differentiation of neurons, cell survival, and synaptic plasticity [22–24]. There are two TrkB receptor isoforms abundantly expressed in the brain; full-length TrkB and truncated TrkB [25]. BDNF activates intracellular signaling cascades through full-length TrkB to induce neuronal proliferation and survival [26], and truncated TrkB regulates extracellular BDNF concentration and activates intracellular signaling pathways [27]. Truncated TrkB induces neurite outgrowth via BDNF-independent mechanism. Although the mechanism is unclear, it seems to be mutually inhibitory with full-length TrkB-mediated outgrowth [28, 29]. Apart from the nervous system, it was also found that TrkB is present in nonnervous cell/tissues, such as myocardial, and pancreatic alpha cells [30, 31].

In mammals, BDNF and TrkB are highly produced and released in several hypothalamus and hippocampus nuclei and known to be involved in glucose and energy homeostases [32–34]. It is well described that the stimulation of the melanocortin-4 receptor (MC_4_-R) decreases food intake and increases energy expenditure [35]. Furthermore, studies showed that BDNF is an important downstream mediator of the melanocortinergic signaling pathway on activation, which is widely expressed in the ventromedial hypothalamus where its expression is regulated by MC_4_-R signaling [35, 36]. All these data may explain the effect of BDNF on food intake, which is reviewed in the following section.

Although BDNF protein was mainly thought to be present in the nervous system, it was also found in endothelial cells, muscle, liver and adipose tissues, and activated immune cells [32, 37–39]. Additionally, BDNF is present in both blood serum and plasma. It has been shown that peripheral BDNF is stored in large amounts in platelets, which are important for storing the BDNF that is secreted from other tissues [40–42]. However, the regulation of BDNF in peripheral blood remains poorly understood.

## 3. BDNF, Food Intake, and Glucose Homeostasis

Regulation of food intake is an important component for energy homeostasis maintenance, which is controlled by different complex mechanisms, including hormonal signaling and multiple molecules. BDNF is one of the key proteins in food intake regulation and body weight control. In 1992, for the first time, Lapchak and Hefti [43] showed that chronic intracerebroventricular infusion of BDNF treatment attenuated weight gain in rats. Another study confirmed the BDNF-induced reduction of food intake [44]. In this study, central injection of BDNF for 3 weeks induced severe, dose-dependent appetite suppression and caused significant body weight loss in rodents and they suggested that appetite suppression may be observed through a hypothalamic serotonergic mechanism.

Besides the key role in food intake regulation, BDNF is significantly related to the regulation of glucose levels. Tonra et al. [10] showed that systemic administration of BDNF decreased nonfasting blood glucose levels without significant reduction in food intake per body weight in obese, non-insulin-dependent diabetic mice with a concomitant decrease in body weight. Similarly, 3 weeks of intermittent BDNF administration significantly reduced blood glucose concentrations and glycated hemoglobin (HbA1c) in *db/db* mice model, which are defined by nonfunctional leptin receptor and provide a model for obesity and non-insulin-dependent T2DM [45]. It was also demonstrated that intracerebroventricular administration of BDNF dose dependently lowers blood glucose to normoglycemic levels and increases pancreatic insulin in *db/db* mice [46]. It is important to note that BDNF does not affect blood glucose in normoglycemic rats [44]. As mentioned above, TrkB receptor was found also in nonnervous cells. Hanyu et al. detected TrkB in mouse islet of Langerhans, and they showed that BDNF downregulates glucagon secretion from mouse pancreatic alpha cells through TrkB and may reduce plasma glucose levels. It suggests that BDNF affects glucose metabolism not only in central metabolic pathways but also in peripheral glucagon secretion pathway [47].

Human studies also showed the key role of BNDF in energy homeostasis [48, 49]. Yeo et al. [48] observed a decreased function of human TrkB receptor with a de novo missense mutation, which causes severe obesity, and this mutation is related to both BDNF and neurotrophin-4/5 [49]. Additionally, decreased BDNF levels in women with low insulin sensitivity compared to high insulin sensitivity group and positive correlation of BDNF with insulin sensitivity were observed. Furthermore, in group with low insulin sensitivity, an association between serum BDNF and adiponectin was also described [50]. These results suggest that BDNF may enhance the energy expenditure, ameliorate systemic glucose balance, and improve insulin sensitivity, and it may be useful in the prevention and management of T2DM.

## 4. T2DM and BDNF

As it is described above, BDNF treatment in obese and diabetic rodents significantly suppressed the blood glucose level, attenuated body weight gain and food intake, and enhanced the energy and glucose metabolism. Both animal experiments and clinical research have shown that BDNF plays a major key role in T2DM [9, 51, 52].

Krabbe et al. [53] observed decreased plasma levels of BDNF in 233 patients with T2DM compared to nondiabetic subjects. Moreover, BDNF was inversely correlated with fasting plasma glucose and homeostasis model assessment of insulin resistance score (HOMA2-IR), which is an indirect measure of insulin resistance. It is possible that circulating levels of BDNF are negatively regulated in response to plasma glucose levels. Recently, similar studies report that median serum levels of BDNF were significantly lower in Chinese T2DM patients compared to healthy controls [54–56]. Li et al. [54] found that lower levels of serum BDNF were negatively correlated with body mass index (BMI) and homeostatic model assessment insulin resistance (HOMA-IR). They also found inverse relation of serum BDNF level to fasting glucose and duration of illness. Passaro et al. [57] investigated the relationship between BDNF, T2DM, and dementia. They noted significantly lower plasma BDNF levels in patient group with both T2DM and dementia than in nondiabetic patients with dementia. Additionally, they also found lower but nonsignificant plasma BDNF levels in 10 patients with only T2DM than in 60 control subjects. Nonsignificant results can be the result of limited number of patients. Similarly, Fujinami et al. [58] found lower serum BDNF levels in 112 T2DM patients compared to heathy subjects. Interestingly, serum BDNF levels of female patients were significantly higher than those of male patients. Also, serum BDNF was positively correlated with immunoreactive insulin and HOMA-IR in female patients. On the other hand, no association was found between BDNF and clinical parameters in male patients. They stated that lack of significance can be due to estrogen and humoral variations. Increasing evidence on neuronal physiology demonstrates a connection between BDNF and steroid hormones such as estradiol and progesterone. Many studies report that estrogen modulates BDNF protein expression [59–62]. Recently, Yi et al. [59] treated the ovariectomised mice with estradiol for 10 days and compared these mice to those ovariectomised ones injected with the vehicle or control group. Estradiol-treated mice showed increased BDNF expression in comparison to group with vehicle injection. In addition to this, it was showed that estradiol injection conjugates protected neurons against global cerebral ischemia and significantly increased BDNF expression on ovariectomised female mice [63]. It is also well described that sex hormones play a key role in glucose metabolism and have significant impact on the development of insulin resistance and T2DM [64]. These findings on sex hormones may explain gender difference of BDNF levels in T2DM patients.

In contrary, Suwa et al. [9] found increased serum BDNF level in 24 female patients with newly diagnosed T2DM who had not previously received either any medication or intervention therapy in comparison to control subjects. In their study, patients with T2DM showed significant positive correlation between serum BDNF level and BMI, percentage of body fat, subcutaneous fat area, triglyceride level, fasting glucose blood level, and HOMA-IR. Another similar results were observed by Boyuk et al. [52]. They reported significantly higher serum levels of BDNF in 88 patients with previously established T2DM in comparison to 33 healthy controls. They also found a positive correlation of HOMA-IR and triglyceride levels with BDNF [52]. As discussed by Boyuk et al., duration of T2DM alone could not explain the elevated BDNF levels. Clinical characteristics of patients and controls, such as gender, antidiabetic and antilipid treatments, smoking history, and obesity conditions, may also have influence on altered BDNF levels (Table 1).

## 5. T2DM, BDNF, and Inflammation

Inflammation plays a key role in insulin resistance and T2DM [65]. Low-grade inflammation has been described as a risk factor of future development of T2DM. Lifestyle alteration and medical treatment decrease the inflammatory condition, which may reduce the risk of T2DM [66]. The mechanism of low-grade inflammation in T2DM is not yet fully understood. However, studies clearly demonstrated that elements of the immune system are altered in obesity and T2DM. The most significant changes take place in adipose tissue, liver, and circulating leukocytes as well as specific cytokines and chemokines [65]. Many studies defined the increased levels of interleukin-1*β* (IL-1*β*), IL-6, and acute phase markers, such as c-reactive protein (CRP), which are predictive components in patients with T2DM [67, 68]. The liver and adipose tissue are important sites for the activation of inflammation pathways. Hepatocytes and myeloid cells such as macrophages induce the production of proinflammatory cytokines, including tumour necrosis factor (TNF), IL-1*β*, and IL-6 [69].

The role of IL-6 in insulin resistance has been contradictory [70]. However, it was shown that increased levels of IL-6 predict future risk of T2DM development [69, 71]. It has an impact on glucose homeostasis, developing obesity, and T2DM including cerebral centers involved in the regulation of energy expenditure and the hypothalamic-pituitary-adrenal axis [72, 73]. Early human study reported that single peripheral administration of IL-6 enhanced energy expenditure in healthy volunteers [74]. In the last decades, it was showed that IL-6-deficient mice developed mature-onset obesity with impaired glucose tolerance and increased glucose levels [75]. Obesity in IL-6-deficient mice was partly reversed by long-term IL-6 replacement. Therefore, in order to understand the possible mechanism and antiobesity effect of IL-6, rodents were administered centrally and peripherally with IL-6. Central IL-6 treatment increased energy expenditure. It may suggest that the antiobesity effect of IL-6 may be exerted at the level of the CNS [75]. IL-6 levels in cerebrospinal fluid were negatively correlated with total body weight and subcutaneous and total body fat in obese humans [76]. Also, the cerebrospinal fluid levels are similar to serum levels and in some individuals could be even higher than in serum [72]. Thus, this evidence suggests that IL-6 affects centers in the CNS, involved in energy regulation and expenditure.

**Figure 1:**
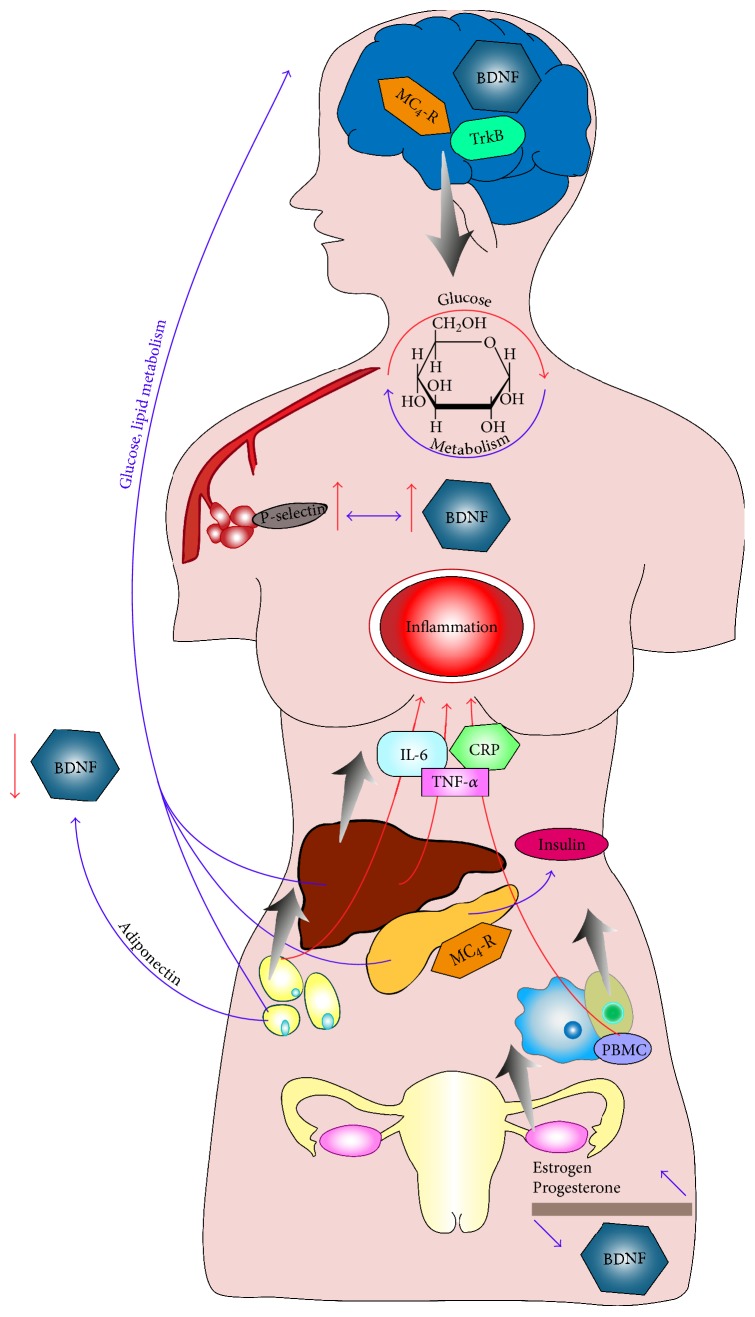
Brain-derived neurotrophic factor (BDNF) and its role on glucose haemostasis and potential relation with inflammation and type 2 diabetes mellitus (T2DM). MC_4_-R: melanocortin 4 receptor; TrkB: tropomyosin receptor kinase B; IL-6: interleukin 6; TNF-*α*: tumor necrosis factor alpha; CRP: c-reactive protein; PBMC: peripheral blood mononuclear cell.

Many studies demonstrated an association of plasma BDNF level with inflammatory conditions [77, 78]. In our study, we observed that IL-6 is associated with increased BDNF concentration in T2DM, which is in accordance with the previous studies, that described similar observations in different clinical conditions [51, 79–81]. In activated antigen-specific T cells and B cells, monocytes produce BDNF and IL-6 and TNF-*α* represents a specific link between monocyte infiltration and neuronal changes in inflammatory diseases [82, 83]. Similarly, Huang et al. [83] found that, in obese individuals comparing to nonobese individuals, peripheral blood mononuclear cells produce a greater amount of BDNF, which is associated with an increased IL-6 response ex vivo. Thus, they suggest that due to increased inflammatory condition, peripheral blood mononuclear cells indicate BDNF and IL-6 expression, which may play a collaborative neuroprotective effect associated with obesity. Additionally, as it was described previously, the possible effect of IL-6 in CNS involved in energy regulation and expenditure has an association with BDNF protein via its important role in the development of the nervous system (Figure 1).

Moreover, plasma BDNF level was also positively correlated with inflammatory cytokines such as high-sensitivity c-reactive protein (hs-CRP) and interferon gamma (IFN-*γ*) in hemodialysis patients. This observation may suggest that plasma BDNF reflects the uremic inflammatory condition in the patients undergoing hemodialysis [80]. Another study showed a positive correlation between serum BDNF levels and white blood cell (WBC) in diabetic patients. Also, BDNF level and CRP were independently associated with T2DM [52]. Additionally, Krabbe et al. [53] found a significant correlation between decreased BDNF concentration and CRP in T2DM independently of obesity. Despite this, no association was found between BDNF and CRP in participants without T2DM. With controversy, recent study showed negative correlation between serum BDNF levels and hs-CRP in patients with T2DM [54].

Peripheral BDNF is stored in large amounts in platelets, and plasma BDNF concentration can be attributed to its release into the plasma from platelets through activation or clotting process [41, 42, 84]. Thus, previous studies investigated the impact of different antiplatelet drugs on BDNF concentrations in both serum and plasma and on the release of BDNF from platelets in healthy volunteers [85]. T2DM is a hypercoagulable state and is associated with platelet hyperreactivity [86]. The etiology of high-platelet reactivity is complex and is related to metabolic disturbances, hyperglycemia, and coexisting inflammation [87, 88]. In particular, inflammatory and coagulation markers have higher concentrations, and platelet reactivity increased in T2DM patients in comparison to healthy subjects [89, 90]. It was described that serum BDNF levels were higher in patients with myocardial infarction and were correlated with P-selectin, which is a biomarker of platelet activation and inflammation [91]. Besides the relation of cardiovascular disease with P-selectin, many studies showed the association between T2DM and platelet hyperactivity due to increased P-selectin expression [92, 93]. Plasma levels of CD40L, IL-6, and P-selectin were significantly higher in patients with T2DM than in control subjects [94, 95]. It was demonstrated that P-selectin concentration is predictive of high-serum BDNF levels in T2DM patients [51]. Recently, an interesting study was performed by Furukawa et al. [96]. They focused on advanced glycation end products (AGEs), which are expressed in human platelets and elevated in patients with T2DM and have adverse effects on cardiovascular functions [97]. The aim of this study was to elucidate the role of AGEs in the regulation of BDNF release from human platelets. They hypothesized that AGE-induced BDNF release is a biological defense system in the early phase of diabetes, and chronic elevation of AGEs may induce depletion or downregulation of BDNF in platelets during the progression of T2DM [96].

Taken together, these data indicate that chronic inflammatory state, enhanced immune system, altered circulating inflammatory cytokines, and elevated compounds released by platelets are associated with BDNF expression that needs to be confirmed with future studies.

## 6. BDNF, Adiponectin, Leptin, and T2DM

The increased risk of T2DM in obesity can partly be described by altered function of adipose tissue, which is a major endocrine organ that secretes a number of active adipocytokines such as leptin, resistin, and adiponectin. They are considered to take part in the regulation of hemostasis, lipid, glucose metabolism, and inflammation [98–100]. Leptin is a peptide hormone, and it acts on the hypothalamus, leading to decreased appetite and increased energy expenditure, thereby modulating metabolism and body weight [101].

Besides the insulin and glucose, it has been reported that the BDNF expression in hypothalamus is regulated by leptin [102]. Studies showed that BDNF administration ameliorates hyperphagia and hyperglycemia in a leptin receptor-deficient *db/db* animal model [10, 45, 103, 104]. It might be speculated that BDNF plays a role in leptin-resistance obesity and T2DM. Maekawa et al. [105] have demonstrated that low BDNF expression in the ventromedial hypothalamus is associated with blood glucose level, increased leptin secretion, and visceral fat mass in T2DM rat model. In their study, administration of BDNF significantly decreased plasma leptin levels in a long-lasting manner concurrently with feeding suppression in T2DM rats with hyperleptinemia. Additionally, Nakagawa et al. [106] reported that repetitive administration of BDNF significantly decreased serum leptin concentration in mice with diet-induced obesity compared with the vehicle-treated group. Additionally, human studies also showed an inverse correlation between BDNF and adiponectin and positive correlation between BDNF and leptin [58, 107–110]. Above mentioned findings indicate that BDNF and leptin may play important roles in the central regulation of energy metabolism and dysregulation of the NT signaling result in obesity.

## 7. BDNF, T2DM, and Antidiabetic Drugs

T2DM is characterized by hyperglycemia resulting from impaired insulin secretion, defects in insulin action, increased hepatic glucose production, and decreased peripheral glucose utilization [1]. A number of drug treatments are available for T2DM therapy.

There are some studies that showed the alteration of BDNF levels on antidiabetic drugs in *in vitro*, animal and human models [111–115]. Yoo et al. [112] showed the relation between BDNF levels and metformin alone and combined with glimepiride treatment in mice. Glimepiride is a second-generation sulfonylurea that increases endogenous insulin secretion from pancreatic beta cells via binding to specific sulfonylurea receptors and induces the increased activity of intracellular insulin receptors. It reduces HbA1c and fasting glucose concentrations. However, body weight gain and hypoglycemia are common adverse effects of all sulfonylureas [116]. Metformin, a biguanide antidiabetic drug, reduces hepatic glucose production and absorption of glucose from gastrointestinal tract and increases insulin uptake in the periphery, enhancing insulin sensitivity in hepatic and peripheral tissues. It decreases HbA1c and fasting glucose concentrations as well as plasma triglyceride and low-density lipoprotein (LDL) cholesterol levels. Metformin treatment has been related to a lack of body weight gain and even weight loss in some overweight patients, contrary to the effects of the sulfonylureas [117]. Besides the antidiabetic effects, retrospective analysis of clinical data suggests that treatment of metformin may reduce cardiovascular morbidity and mortality [118, 119]. In an animal model, after 5 weeks of the high-fat diet, metformin alone or metformin with glimepiride was administered orally once a day for 3 weeks. Metformin alone or metformin with glimepiride treatment resulted in a decrease weight gain and food intake. Furthermore, BDNF protein levels were significantly reduced with the high-fat diet + vehicle-treated group in the dentate gyrus, which is an input region of the hippocampus compared to the low-fat diet-treated group. The administration of metformin or metformin with glimepiride in high-fat diet group prevented the reduction of BDNF levels in the dentate gyrus [112]. Another similar study showed the significantly higher BDNF levels in metformin-treated group than both control group and the group with 1-methyl-4-phenyl-1,2,3,6-tetrahydropyridine- (MPTP-) induced Parkinsonism in mice [113]. Additionally, Hristova found nonsignificantly higher plasma BDNF levels in patients treated with metformin [111]. On the other hand, lower BDNF mRNA levels were found after the administration of metformin than the control group in mice; however, no significant BDNF protein alteration was observed [114].

In order to understand the impact of metformin on BDNF levels, Ma et al. [115] investigated the effect of metformin on Schwann cells under hypoxia condition and they found that the mRNA levels of BDNF were significantly decreased. However, this detrimental effect of hypoxia on gene expression in Schwann cells was partially reversed by metformin. The mRNA level of BDNF in metformin-treated Schwann cells was higher than those without metformin under hypoxia condition. This beneficial effect of metformin on gene expression under hypoxia condition was significantly inhibited by compound C, which is an inhibitor of AMP-activated protein kinase (AMPK) and an important cellular regulator of lipid and glucose metabolism [115, 120]. It was defined in many studies that the lipid-lowering effect of metformin is largely attributed to the activation of the energy sensor-AMPK in hepatocytes [121–124] and can suppress de novo lipogenesis in hepatocytes by activating AMPK [125]. Additionally, in order to confirm the association between metformin and BDNF levels, Yoo et al. [112] infused K252a, a potent TrkB and TrkC receptor inhibitor to mice. Injection of K252a to the metformin-treated group significantly reversed the amelioration of the reduction of neuroblast differentiation induced by high-fat diet.

Taken all together, these findings suggest that the correlation between BDNF and metformin may be the reason of metformin-induced insulin action by insulin receptor binding, metformin-induced high BDNF levels due to increasing AMPK, and enhanced tyrosine kinase receptor activity which may amplify BDNF signaling [126–128].

## 8. Delivery of NTs to the CNS

As mentioned above, BDNF is the most abundant NT and a regulator of neuronal differentiation and survival in the mammalian CNS, and it is a key molecular target in the development of drugs against neurodegenerative disorders [14–16]. Several studies showed the peripheral administration of BDNF both in human and animal models; however, BDNF is a moderately sized protein and a small amount of it can cross the blood-brain barrier (BBB) [129]. Therefore, the effect on CNS is lower than expected, and for this reason, previous researchers focused on the administration of BDNF directly into the CNS.

As previously reviewed by Géral et al., the first clinical trials have been performed using subcutaneous or intrathecal administrations of recombinant human BDNF in patients with amyotrophic lateral sclerosis (a motor neuron degenerative disease) [130]. The BDNF treatment has been well tolerated, but it has lacked efficacy, due to very short *in vivo* half-life therapeutic protein (<2 min) and its limited penetration through the BBB [130, 131]. Besides BDNF, other NTs such as glial cell-derived neurotrophic factor (GDNF) was studied. Some researches analyzed intrathecal infusion of GDNF into CNS in patients with Parkinson's disease; however, the results were inconclusive since low flow rates of infusion failed to show efficacy and achieved low distribution of GDNF to the target region. Addition to this, several side effects were observed including nausea, loss of appetite, depression, and hallucination probably due to potential tissue damage and local side effects in the area of the injections. Hence, contrary to expectations, intraventricular and/or intrathecal administrations of NTs resulted in little penetration of the brain and/or spinal cord parenchyma [132].

Besides neurodegenerative disorders, some studies also focused on intrathecal injection of BDNF in T2DM, yet the number is limited and only based on neuropathic pain caused by diabetes in an animal model. Recently, Li et al. [133] aimed to investigate the role of exogenous BDNF and its high-affinity TrkB in rats with streptozotocin-induced diabetic neuropathic pain. They revealed that continued intrathecal administration of BDNF to diabetic rats dramatically alleviated mechanical and thermal hyperalgesia, as well as inhibited hyperexcitability of dorsal root ganglion (DRG) neurons, and these effects were blocked by pretreatment with TrkB/Fc (a synthetic fusion protein consisting of the extracellular ligand-binding domain of the TrkB receptor, BDNF sequester). Thus, they showed that intrathecal BDNF treatment relieved pain symptoms of diabetic rats by reducing hyperexcitability of DRG neurons. Another study demonstrated that the microglial activation at the spinal cord contributes to mechanical hyperalgesia and spinal neuronal hyperactivity induced by diabetes, by reducing the potassium chloride cotransporter 2 expression. Blocking the BDNF action in streptozotocin-induced diabetic rats by TrkB/Fc, crystallizable domain was found to induce moderate effects on mechanical hyperalgesia, although BDNF levels were not increased in streptozotocin-induced diabetic rats [134]. In addition to neuropathies, it was also reported that BDNF had beneficial effects on diabetic retinopathy. Retinal levels of BDNF are reduced in animal models of streptozotocin-induced diabetes, and this reduction of BDNF is correlated with amacrine cell degeneration. Intraocular administration of BDNF to diabetic rats rescued dopaminergic amacrine cells from cell degeneration [135].

### 8.1. Gene Delivery to the Brain, a New Perspective

Since intrathecal administrations of NTs resulted in little penetration of the brain or spinal cord parenchyma, recent studies tried NGF and BDNF gene deliveries via vehicles. Zheng et al. [136] aimed to investigate the potential role of NGF and the involvement of TRPV1 receptor in isolated diabetic mouse hearts following ischemia/reperfusion injury, as NGF plays an essential role in diabetic neuropathy and ischemic heart disease. They used adenovirus as a vector to deliver NGF gene on streptozotocin-induced diabetic mice hearts. They showed that NGF improved cardiac performance and promoted prosurvival in ischemic cardiomyocytes and diabetic-isolated hearts. Walwyn et al. [137] delivered the NGF gene via a herpes simplex viral vector to dorsal root ganglion neurons and delayed the development of hypoalgesia in a leptin receptor mutant mouse, which is an evidence that NGF can restore myelinated nerve fiber morphology.

## 9. Can BDNF Treat T2DM?

Fetal or early postnatal depletion of BDNF or its receptor, TrkB, in rodents results in hyperphagia and severe obesity [35, 138–140]. BDNF is an important downstream mediator of MC_4_-R signaling, and its administration regulates the obesity in MC_4_-R knockout mice [35]. Besides the effects on obesity, BDNF prevents the development of T2DM in prediabetic *db/db* mice as we discussed above [11]. Administration of BDNF suppressed food intake with a concomitant body weight loss, and decreased serum HbA1c insulin and glucose levels in diabetic rodents suggest that BDNF treatment improves insulin sensitivity [10, 44, 50, 104, 124]. Also, it reduced glucagon secretion in isolated mice pancreatic islets, without affecting insulin secretion [47]. Moreover, BDNF treatment regulates glucose metabolism and inhibits pancreatic exhaustion in obese T2DM mice [141]. However, when BDNF is injected to normoglycemic rodents, it has no effect on blood glucose levels [45].

Some studies compared the antidiabetic effects of BDNF and standard antidiabetic drugs. Yamanaka et al. [141] treat the obese diabetic mice with BDNF or thiazolidinediones. BDNF significantly and more effectively decreased the food intake than pioglitazone and rosiglitazone. Compared with thiazolidinediones, BDNF more potently ameliorates pancreatic dysfunction, fatty liver, and energy expenditure, thereby exerting favourable antidiabetic effects in diabetic mice. Thus, authors suggest that exogenous BDNF administration shows its antidiabetic and antilipidemic effects similar to thiazolidinediones.

These data suggest that BDNF might be used as a potential antidiabetic treatment; however, both peripheral and intrathecal administration of BNDF caused adverse side effects. For example, some studies showed that BDNF infusion lowered sensory thresholds and increased pain in animal model [142]. Importantly, BDNF/TrkB signaling has been reported to be associated with tumor progression and metastasis in several human malignancies, such as multiple myeloma and breast tumor [143, 144].

## 10. Conclusion

Taken together, we concluded that BDNF may enhance the energy expenditure, ameliorate systemic glucose balance, and improve insulin sensitivity, and it may be useful in the prevention and management of T2DM. Different studies reported the antidiabetic and antilipidemic effects of BDNF. However, abovementioned limitations of BDNF administration due to unsuitable pharmacokinetic profiles (i.e., poor BBB penetrability, short half-lives, and low bioavailability) and previous unsuccessful experiences question currently its clinical utilization. Additionally, a route of administration of BDNF caused serious side effects, and intraventricular/intrathecal injections failed to provide reliable effects. Apart from this, chronic inflammatory state, enhanced immune system, and altered circulating inflammatory cytokines are associated with BDNF expression. So far, animal studies mostly focused on the modulation of blood glucose and insulin levels after BDNF administration; however, there is no study to investigate whether BDNF treatment has an influence on alteration of inflammatory markers in T2DM. Thus, future studies of BDNF-induced inflammatory regulation in diabetes are needed.

## Figures and Tables

**Table 1:** 

Publication	Ethnic and duration of T2DM	Material/criteria	Number of T2DM patients/control	Characteristics of the T2DM patients	Alteration of BDNF level with T2DM	Positive-correlated parameters with BDNF level	Negative-correlated parameters with BDNF level	Independent association with BNDF level
Suwa et al. [9] 2006	Japanese female, newly diagnosed	(i) Serum (ii) No treatment	24/7	(i) BMI (kg/m^2^): 26.1 ± 3.3 (ii) HbA1c (%): 6.52 ± 0.90 (iii) HOMA-IR: 2.32 ± 1.38	**↑** Increased	(i) BMI (ii) Body fat percent (iii) Subcutaneous fat (iv) HOMA-IR (v) Triglycerides (vi) Fasting glucose (vii) HbA1c (weak correlation)	(i) Age	

Krabbe et al. [53] 2007	Caucasian, long standing	(i) Plasma (ii) No treatment with insulin (iii) No antidiabetic drug for 1 week before the blood sampling (iv) No drug 24 hours before the blood sampling	Diabetes obese/obese: 46/41 Diabetes nonobese/nonobese: 50/62	Diabetes obese patients: (i) BMI (kg/m^2^): 35.5 ± 0.7 (ii) Fasting glucose (mmol/L): 9.6 ± 0.9 Diabetes nonobese patients: (iii) BMI (kg/m^2^): 26.6 ± 0.3 (iv) Fasting glucose (mmol/L): 9.9 ± 0.6	**↓** Decreased with patient with diabetes compared to nondiabetic **↓** Decreased with obese patients compared to nonobese	(i) CRP	(i) Fasting glucose level (strong correlation)	(i) Obesity

Fujinami et al. [58] 2008	Japanese, long standing	(i) Serum (ii) No treatment with insulin (iii) No treatment with thiazolidinedione (iv) No hormonal treatment	112/80	(i) BMI (kg/m^2^): 23.1 ± 3.1 (ii) Fasting glucose (mmol/L): 8.51 ± 2.18 (iii) HbA1c (%): 7.4 ± 1.3 (iv) HOMA-R: 2.42 ± 2.16	**↓** Decreased **↑** Increased female patients with diabetes compared to male patients with diabetes	(i) Immunoreactive insulin (ii) HOMA-R	(i) Adiponectin level (weak correlation)	

Zhen et al. [56] 2013	Han Chinese, long standing	(i) Serum (ii) No treatment with insulin (iii) Some patients received metformin and/or repaglinide	208/212	(i) BMI (kg/m^2^): 25.9 ± 3.8 (ii) Fasting glucose (mmol/L): 9.2 ± 3.1 (iii) HbA1c (%): 7.5 ± 2.0	**↓** Decreased	(i) Delayed memory index (ii) Cognitive deficit	(i) Fasting glucose level (ii) Duration of illness	

He et al. 2014 [55] (pilot study)	Chinese, long standing	(i) Serum (ii) No data	37/37	(i) BMI (kg/m^2^): 26.4 ± 2.3 (ii) Fasting glucose (mmol/L): 8.37 ± 3.39 (iii) HbA1c (%): 9.11 ± 2.92	**↓** Decreased **↑** Increased female patients with diabetes compared to male patients with diabetes			

Passaro et al. [57] 2014	Caucasian, long standing	(i) Plasma (ii) Some patients received antidiabetic drug (iii) Antihypertension drug	37/154		**↓** Decreased	(i) Total cholesterol (ii) LDL	(i) Age (ii) Systolic blood pressure	(i) T2DM

Boyuk et al. [52] 2014	Caucasian, long standing	(i) Serum (ii) Some patients received oral antidiabetic drug (iii) Oral antidiabetic drug plus insulin (iv) Insulin (v) Antihypertension drug (vi) Antilipid drug	88/33	(i) BMI (kg/m^2^): 31.52 ± 5.80 (ii) Fasting glucose (mg/dL): 170.36 ± 91.21 (iii) HbA1c (%): 8.30 ± 2.37 (iv) HOMA-IR: 3.73 ± 3.05	**↑** Increased	(i) HOMA-IR (ii) Triglyceride (iii) WBC		(i) T2DM

Li et al. [54] 2015	Chinese, long standing	(i) Serum (ii) Some patients received insulin (iii) Lipid-lowering medication (iv) Blood pressure treatment	292/200	(i) BMI (kg/m^2^): 26.8 (24.7–29.4) (ii) Fasting glucose (mmol/L): 8.6 (6.7–9.9) (iii) HbA1c (%): 7.9 (6.4–9.2) (iv) HOMA-IR: 3.80 (2.35–5.47)	**↓** Decreased		(i) Fasting glucose level (ii) Duration of illness (iii) BMI (iv) CRP	

BMI: body mass index; HbA1c: glycated hemoglobin; HOMA-IR: homeostatic model assessment insulin resistance; CRP: c-reactive protein; LDL: low-density lipoprotein.

## References

[1] American Diabetes Association (2009). Diagnosis and classification of diabetes mellitus. *Diabetes Care*.

[2] Lontchi-Yimagou E., Sobngwi E., Matsha T. E., Kengne A. P. (2013). Diabetes mellitus and inflammation. *Current Diabetes Reports*.

[3] Mattson M. P., Maudsley S., Martin B. (2004). BDNF and 5-HT: a dynamic duo in age-related neuronal plasticity and neurodegenerative disorders. *Trends in Neurosciences*.

[4] Cohen-Cory S., Kidane A. H., Shirkey N. J., Marshak S. (2010). Brain-derived neurotrophic factor and the development of structural neuronal connectivity. *Developmental Neurobiology*.

[5] Mirowska-Guzel D. (2009). The role of neurotrophic factors in the pathology and treatment of multiple sclerosis. *Immunopharmacology and Immunotoxicology*.

[6] Gezen-Ak D., Dursun E., Hanağası H. (2013). BDNF, TNF*α*, HSP90, CFH, and IL-10 serum levels in patients with early or late onset Alzheimer’s disease or mild cognitive impairment. *Journal of Alzheimer's Disease*.

[7] Nagahara A. H., Tuszynski M. H. (2011). Potential therapeutic uses of BDNF in neurological and psychiatric disorders. *Nature Reviews Drug Discovery*.

[8] Manni L., Nikolova V., Vyagova D., Chaldakov G. N., Aloe L. (2005). Reduced plasma levels of NGF and BDNF in patients with acute coronary syndromes. *International Journal of Cardiology*.

[9] Suwa M., Kishimoto H., Nofuji Y. (2006). Serum brain-derived neurotrophic factor level is increased and associated with obesity in newly diagnosed female patients with type 2 diabetes mellitus. *Metabolism*.

[10] Tonra J. R., Ono M., Liu X. (1999). Brain-derived neurotrophic factor improves blood glucose control and alleviates fasting hyperglycemia in C57BLKS-Lepr (db)/lepr(db) mice. *Diabetes*.

[11] Yamanaka M., Itakura Y., Tsuchida A., Nakagawa T., Taiji M. (2008). Brain-derived neurotrophic factor (BDNF) prevents the development of diabetes in prediabetic mice. *Biomedical Research*.

[12] Reichardt L. F. (2006). Neurotrophin-regulated signalling pathways. *Philosophical Transactions of the Royal Society of London, Series B: Biological Sciences*.

[13] Chao M. V., Rajagopal R., Lee F. S. (2006). Neurotrophin signalling in health and disease. *Clinical Science (London)*.

[14] Farinas I. (1999). Neurotrophin actions during the development of the peripheral nervous system. *Microscopy Research and Technique*.

[15] McAllister A. K. (2001). Neurotrophins and neuronal differentiation in the central nervous system. *Cellular and Molecular Life Sciences*.

[16] Cohen S., Greenberg M. E. (2008). Communication between the synapse and the nucleus in neuronal development, plasticity, and disease. *Annual Review of Cell and Developmental Biology*.

[17] Rodriguez-Tebar A., Dechant G., Barde Y. A. (1990). Binding of brain-derived neurotrophic factor to the nerve growth factor receptor. *Neuron*.

[18] Klein R., Nanduri V., Jing S. A. (1991). The trkB tyrosine protein kinase is a receptor for brain-derived neurotrophic factor and neurotrophin-3. *Cell*.

[19] Bartkowska K., Turlejski K., Djavadian R. L. (2010). Neurotrophins and their receptors in early development of the mammalian nervous system. *Acta Neurobiologiae Experimentalis (Wars)*.

[20] Lee R., Kermani P., Teng K. K., Hempstead B. L. (2001). Regulation of cell survival by secreted proneurotrophins. *Science*.

[21] Friedman W. J. (2000). Neurotrophins induce death of hippocampal neurons via the p75 receptor. *Journal of Neuroscience*.

[22] Hofer M. M., Barde Y. A. (1988). Brain-derived neurotrophic factor prevents neuronal death in vivo. *Nature*.

[23] Knüsel B., Winslow J. W., Rosenthal A. (1991). Promotion of central cholinergic and dopaminergic neuron differentiation by brainderived neurotrophic factor but not neurotrophin 3. *Proceedings of the National Academy of Sciences of the United States of America*.

[24] Cowansage K. K., LeDoux J. E., Monfils M. H. (2010). Brain-derived neurotrophic factor: a dynamic gatekeeper of neural plasticity. *Current Molecular Pharmacology*.

[25] Klein R., Conway D., Parada L. F., Barbacid M. (1990). The trkB tyrosine protein kinase gene codes for a second neurogenic receptor that lacks the catalytic kinase domain. *Cell*.

[26] Numakawa T., Suzuki S., Kumamaru E., Adachi N., Richards M., Kunugi H. (2010). BDNF function and intracellular signaling in neurons. *Histology and Histopathology*.

[27] Ohira K., Homma K. J., Hirai H., Nakamura S., Hayashi M. (2006). TrkB-T1 regulates the RhoA signaling and actin cytoskeleton in glioma cells. *Biochemical and Biophysical Research Communications*.

[28] Yacoubian T. A., Lo D. C. (2000). Truncated and full-length TrkB receptors regulate distinct modes of dendritic growth. *Nature Neuroscience*.

[29] Fryer R. H., Kaplan D. R., Kromer L. F. (1997). Truncated trkB receptors on nonneuronal cells inhibit BDNF-induced neurite outgrowth in vitro. *Experimental Neurology*.

[30] Shibayama E., Koizumi H. (1996). Cellular localization of the Trk neurotrophin receptor family in human non-neuronal tissues. *American Journal of Pathology*.

[31] Takei N., Furukawa K., Hanyu O., Sone H., Nawa H. (2014). A possible link between BDNF and mTOR in control of food intake. *Frontiers in Psychology*.

[32] Noble E. E., Billington C. J., Kotz C. M., Wang C. (2011). The lighter side of BDNF. *American Journal of Physiology-Regulatory, Integrative and Comparative Physiology*.

[33] Vanevski F., Xu B. (2013). Molecular and neural bases underlying roles of BDNF in the control of body weight. *Frontiers in Neuroscience*.

[34] Rothman S. M., Griffioen K. J., Wan R., Mattson M. P. (2012). Brain-derived neurotrophic factor as a regulator of systemic and brain energy metabolism and cardiovascular health. *Annals of the New York Academy of Sciences*.

[35] Xu B., Goulding E. H., Zang K. (2003). Brain-derived neurotrophic factor regulates energy balance downstream of melanocortin-4 receptor. *Nature Neuroscience*.

[36] Bariohay B., Roux J., Tardivel C., Trouslard J., Jean A., Lebrun B. (2009). Brain-derived neurotrophic factor/tropomyosin-related kinase receptor type B signaling is a downstream effector of the brainstem melanocortin system in food intake control. *Endocrinology*.

[37] Donovan M. J., Lin M. I., Wiegn P. (2000). Brain derived neurotrophic factor is an endothelial cell survival factor required for intramyocardial vessel stabilization. *Development*.

[38] Cassiman D., Denef C., Desmet V. J., Roskams T. (2001). Human and rat hepatic stellate cells express neurotrophins and neurotrophin receptors. *Hepatology*.

[39] Kerschensteiner M., Gallmeier E., Behrens L. (1999). Activated human T cells, B cells, and monocytes produce brain-derived neurotrophic factor in vitro and in inflammatory brain lesions: a neuroprotective role of inflammation?. *The Journal of Experimental Medicine*.

[40] Karege F., Bondolfi G., Gervasoni N., Schwald M., Aubry J. M., Bertschy G. (2005). Low brain-derived neurotrophic factor (BDNF) levels in serum of depressed patients probably results from lowered platelet BDNF release unrelated to platelet reactivity. *Biological Psychiatry*.

[41] Yamamoto H., Gurney M. E. (1990). Human platelets contain brain-derived neurotrophic factor. *Journal of Neuroscience*.

[42] Fujimura H., Altar C. A., Chen R. (2002). Brain-derived neurotrophic factor is stored in human platelets and released by agonist stimulation. *Thrombosis and Haemostasis*.

[43] Lapchak P. A., Hefti F. (1992). BDNF and NGF treatment in lesioned rats: effects on cholinergic function and weight gain. *Neuroreport*.

[44] Pelleymounter M. A., Cullen M. J., Wellman C. L. (1995). Characteristics of BDNF-induced weight loss. *Experimental Neurology*.

[45] Ono M., Itakura Y., Nonomura T. (2000). Intermittent administration of brain-derived neurotrophic factor ameliorates glucose metabolism in obese diabetic mice. *Metabolism*.

[46] Nonomura T., Tsuchida A., Ono-Kishino M., Nakagawa T., Taiji M., Noguchi H. (2001). Brain-derived neurotrophic factor regulates energy expenditure through the central nervous system in obese diabetic mice. *International Journal of Experimental Diabetes Research*.

[47] Hanyu O., Yamatani K., Ikarashi T. (2003). Brain-derived neurotrophic factor modulates glucagon secretion from pancreatic alpha cells: its contribution to glucose metabolism. *Diabetes, Obesity and Metabolism*.

[48] Yeo G. S., Connie Hung C. C., Rochford J. (2004). A de novo mutation affecting human TrkB associated with severe obesity and developmental delay. *Nature Neuroscience*.

[49] Gray J., Yeo G. S., Cox J. J. (2006). Hyperphagia, severe obesity, impaired cognitive function, and hyperactivity associated with functional loss of one copy of the brain-derived neurotrophic factor (BDNF) gene. *Diabetes*.

[50] Karczewska-Kupczewska M., Kowalska I., Nikołajuk A. (2012). Circulating brain-derived neurotrophic factor concentration is downregulated by intralipid/heparin infusion or high-fat meal in young healthy male subjects. *Diabetes Care*.

[51] Eyileten C., Zaremba M., Janicki P. K. (2016). Serum brain-derived neurotrophic factor is related to platelet reactivity but not to genetic polymorphisms within BDNF encoding gene in patients with type 2 diabetes. *Medical Science Monitor*.

[52] Boyuk B., Degirmencioglu S., Atalay H. (2014). Relationship between levels of brain-derived neurotrophic factor and metabolic parameters in patients with type 2 diabetes mellitus. *Journal of Diabetes Research*.

[53] Krabbe K. S., Nielsen A. R., Krogh-Madsen R. (2007). Brain-derived neurotrophic factor (BDNF) and type 2 diabetes. *Diabetologia*.

[54] Li B., Lang N., Cheng Z. F. (2016). Serum levels of brain-derived neurotrophic factor are associated with diabetes risk, complications, and obesity: a cohort study from Chinese patients with type 2 diabetes. *Molecular Neurobiology*.

[55] He M., Wang J. (2014). Decreased serum brain-derived neurotrophic factor in Chinese patients with type 2 diabetes mellitus. *Acta Biochimica et Biophysica Sinica Shanghai*.

[56] Zhen Y. F., Zhang J., Liu X. Y. (2013). Low BDNF is associated with cognitive deficits in patients with type 2 diabetes. *Psychopharmacology*.

[57] Passaro A., Dalla Nora E., Morieri M. L. (2015). Brain-derived neurotrophic factor plasma levels: relationship with dementia and diabetes in the elderly population. *The Journals of Gerontology Series A, Biological Sciences and Medical Sciences*.

[58] Fujinami A., Ohta K., Obayashi H. (2008). Serum brain-derived neurotrophic factor in patients with type 2 diabetes mellitus: relationship to glucose metabolism and biomarkers of insulin resistance. *Clinical Biochemistry*.

[59] Yi H., Bao X., Tang X., Fan X., Xu H. (2016). Estrogen modulation of calretinin and BDNF expression in midbrain dopaminergic neurons of ovariectomised mice. *Journal of Chemical Neuroanatomy*.

[60] Aguirre C. C., Baudry M. (2009). Progesterone reverses 17beta-estradiol-mediated neuroprotection and BDNF induction in cultured hippocampal slices. *European Journal of Neuroscience*.

[61] Singh M., Meyer E., Simpkins J. (1995). The effect of ovariectomy and estradiol replacement on brain derived neurotrophic factor messenger ribonucleic acid expression in cortical and hippocampal brain regions of female Sprague-Dawley rats. *Endocrinology*.

[62] Sohrabji F., Miranda R., Toran-Allerand C. (1995). Identification of a putative estrogen response element in the gene coding for BDNF. *Proceedings of the National Academy of Sciences of the United States of America*.

[63] Numakawa T., Richards M., Nakajima S. (2014). The role of brain-derived neurotrophic factor in comorbid depression: possible linkage with steroid hormones, cytokines, and nutrition. *Frontiers in Psychiatry*.

[64] Comitato R., Saba A., Turrini A., Arganini C., Virgili F. (2015). Sex hormones and macronutrient metabolism. *Critical Reviews in Food Science and Nutrition*.

[65] Shoelson S. E., Lee J., Goldfine A. B. (2006). Inflammation and insulin resistance. *Journal of Clinical Investigation*.

[66] Eizirik D. L., Mandrup-Poulsen T. (2001). A choice of death: the signal-transduction of immune-mediated beta-cell apoptosis. *Diabetologia*.

[67] Spranger J., Kroke A., Möhlig M. (2003). Inflammatory cytokines and the risk to develop type 2 diabetes: results of the prospective population-based European prospective investigation into cancer and nutrition (EPIC)-Potsdam study. *Diabetes*.

[68] Pradhan A. D., Manson J. E., Rifai N., Buring J. E., Ridker P. M. (2001). C-reactive protein, interleukin 6, and risk of developing type 2 diabetes mellitus. *The Journal of the American Medical Association*.

[69] Cai D., Yuan M., Frantz D. F. (2005). Local and systemic insulin resistance resulting from hepatic activation of IKK-beta and NF-kappaB. *Nature Medicine*.

[70] Mohamed-Ali V., Goodrick S., Rawesh A. (1997). Subcutaneous adipose tissue releases interleukin-6, but not tumor necrosis factor-alpha, in vivo. *The Journal of Clinical Endocrinology & Metabolism*.

[71] Hu F. B., Meigs J. B., Li T. Y., Rifai N., Manson J. E. (2004). Inflammatory markers and risk of developing type 2 diabetes in women. *Diabetes*.

[72] Wallenius K., Jansson J. O., Wallenius V. (2003). The therapeutic potential of interleukin-6 in treating obesity. *Expert Opinion on Biological Therapy*.

[73] Kristiansen O. P., Mandrup-Poulsen T. (2005). Interleukin-6 and diabetes: the good, the bad, or the indifferent?. *Diabetes*.

[74] Tsigos C., Papanicolaou D. A., Defensor R., Mitsiadis C. S., Kyrou I., Chrousos G. P. (1997). Dose effects of recombinant human interleukin-6 on pituitary hormone secretion and energy expenditure. *Neuroendocrinology*.

[75] Wallenius V., Wallenius K., Ahrén B. (2002). Interleukin-6-deficient mice develop mature-onset obesity. *Nature Medicine*.

[76] Stenlöf K., Wernstedt I., Fjällman T., Wallenius V., Wallenius K., Jansson J. O. (2003). Interleukin-6 levels in the central nervous system are negatively correlated with fat mass in overweight/obese subjects. *The Journal of Clinical Endocrinology & Metabolism*.

[77] Dicou E., Masson C., Jabbour W., Nerriere V. (1993). Increased frequency of NGF in sera of rheumatoid arthritis and systemic lupus erythematosus patients. *Neuroreport*.

[78] Bonini S., Lambiase A., Angelucci F., Magrini L., Manni L., Aloe L. (1996). Circulating nerve growth factor levels are increased in humans with allergic diseases and asthma. *Proceedings of the National Academy of Sciences of the United States of America*.

[79] Patanella A. K., Zinno M., Quaranta D. (2010). Correlations between peripheral blood mononuclear cell production of BDNF, TNF-alpha, IL-6, IL-10 and cognitive performances in multiple sclerosis patients. *Journal of Neuroscience Research*.

[80] Shin S. J., Yoon H. E., Chung S., Kim Y. G., Kim D. J. (2012). Plasma brain-derived neurotrophic factor in hemodialysis patients. *International Journal of Medical Sciences*.

[81] Patas K., Penninx B. W., Bus B. A. (2014). Association between serum brain-derived neurotrophic factor and plasma interleukin-6 in major depressive disorder with melancholic features. *Brain, Behavior, and Immunity*.

[82] Schulte-Herbrüggen O., Nassenstein C., Lommatzsch M., Quarcoo D., Renz H., Braun A. (2005). Tumor necrosis factor-alpha and interleukin-6 regulate secretion of brain-derived neurotrophic factor in human monocytes. *Journal of Neuroimmunology*.

[83] Huang C. J., Mari D. C., Whitehurst M., Slusher A., Wilson A., Shibata Y. (2014). Brain-derived neurotrophic factor expression ex vivo in obesity. *Physiology & Behavior*.

[84] Pliego-Rivero F. B., Bayatti N., Giannakoulopoulos X. (1997). Brain-derived neurotrophic factor in human platelets. *Biochemical Pharmacology*.

[85] Stoll P., Plessow A., Bratke K., Virchow J. C., Lommatzsch M. (2011). Differential effect of clopidogrel and aspirin on the release of BDNF from platelets. *Journal of Neuroimmunology*.

[86] Yazbek N., Bapat A., Kleiman N. (2003). Platelet abnormalities in diabetes mellitus. *Coronary Artery Disease*.

[87] Coban E., Yazicioglu G., Ozdogan M. (2007). Platelet activation in subjects with subclinical hypothyroidism. *Medical Science Monitor*.

[88] Ferreiro J. L., Gomez-Hospital J. A., Angiolillo D. J. (2010). Platelet abnormalities in diabetes mellitus. *Diabetes & Vascular Disease Research*.

[89] Marx N., Imhof A., Froehlich J. (2003). Effect of rosiglitazone treatment on soluble CD40L in patients with type 2 diabetes and coronary artery disease. *Circulation*.

[90] Gokulakrishnan K., Deepa R., Mohan V., Gross M. D. (2006). Soluble P-selectin and CD40L levels in subjects with prediabetes, diabetes mellitus, and metabolic syndrome: the Chennai urban rural epidemiology study. *Metabolism*.

[91] Lorgis L., Amoureux S., de Maistre E. (2010). Serum brain-derived neurotrophic factor and platelet activation evaluated by soluble P-selectin and soluble CD-40-ligand in patients with acute myocardial infarction. *Fundamental & Clinical Pharmacology*.

[92] Kakouros N., Rade J. J., Kourliouros A., Resar J. R. (2011). Platelet function in patients with diabetes mellitus: from a theoretical to a practical perspective. *International Journal of Endocrinology*.

[93] Schneider D. J., Hardison R. M., Lopes N., Sobel B. E., Brooks M. M. (2009). Pro-thrombosis ancillary study group. Association between increased platelet P-selectin expression and obesity in patients with type 2 diabetes: a BARI 2D (bypass angioplasty revascularization investigation 2 diabetes) substudy. *Diabetes Care*.

[94] Lim H. S., Blann A. D., Lip G. Y. (2004). Soluble CD40 ligand, soluble P-selectin, interleukin-6, and tissue factor in diabetes mellitus: relationships to cardiovascular disease and risk factor intervention. *Circulation*.

[95] Neubauer H., Setiadi P., Günesdogan B., Pinto A., Börgel J., Mügge A. (2010). Influence of glycaemic control on platelet bound CD40-CD40L system, P-selectin and soluble CD40 ligand in type 2 diabetes. *Diabetic Medicine*.

[96] Furukawa K., Fuse I., Iwakura Y. (2017). Advanced glycation end products induce brain-derived neurotrophic factor release from human platelets through the Src-family kinase activation. *Cardiovascular Diabetology*.

[97] Mapanga R. F., Essop M. F. (2016). Damaging effects of hyperglycemia on cardiovascular function: spotlight on glucose metabolic pathways. *American Journal of Physiology Heart and Circulatory Physiology*.

[98] Matsuzawa Y., Funahashi T., Nakamura T. (1999). Molecular mechanism of metabolic syndrome X: contribution of adipocytokines, adipocyte-derived bioactive substances. *Annals of the New York Academy of Sciences*.

[99] Rasouli N., Kern P. A. (2008). Adipocytokines and the metabolic complications of obesity. *The Journal of Clinical Endocrinology & Metabolism*.

[100] Kapłon-Cieślicka A., Postuła M., Rosiak M. (2015). Association of adipokines and inflammatory markers with lipid control in type 2 diabetes. *Polish Archives of Internal Medicine*.

[101] Farooqi I. S., O’Rahilly S. (2009). Leptin: a pivotal regulator of human energy homeostasis. *The American Journal of Clinical Nutrition*.

[102] Komori T., Morikawa Y., Nanjo K., Senba E. (2006). Induction of brain-derived neurotrophic factor by leptin in the ventromedial hypothalamus. *Neuroscience*.

[103] Ono M., Ichihara J., Nonomura T. (1997). Brain-derived neurotrophic factor reduces blood glucose level in obese diabetic mice but not in normal mice. *Biochemical and Biophysical Research Communications*.

[104] Tsuchida A., Nonomura T., Ono-Kishino M., Nakagawa T., Taiji M., Noguchi H. (2001). Acute effects of brain-derived neurotrophic factor on energy expenditure in obese diabetic mice. *International Journal of Obesity and Related Metabolic Disorders*.

[105] Maekawa F., Fujiwara K., Toriya M. (2013). Brain-derived neurotrophic factor in VMH as the causal factor for and therapeutic tool to treat visceral adiposity and hyperleptinemia in type 2 diabetic Goto-Kakizaki rats. *Frontiers in Synaptic Neuroscience*.

[106] Nakagawa T., Ogawa Y., Ebihara K. (2003). Anti-obesity and anti-diabetic effects of brain-derived neurotrophic factor in rodent models of leptin resistance. *International Journal of Obesity and Related Metabolic Disorders*.

[107] Ehrlich S., Salbach-Andrae H., Eckart S. (2009). Serum brain-derived neurotrophic factor and peripheral indicators of the serotonin system in underweight and weight-recovered adolescent girls and women with anorexia nervosa. *Journal of Psychiatry & Neuroscience*.

[108] Golden E., Emiliano A., Maudsley S. (2010). Circulating brain-derived neurotrophic factor and indices of metabolic and cardiovascular health: data from the Baltimore longitudinal study of aging. *PLoS One*.

[109] Roth C. L., Elfers C., Gebhardt U., Müller H. L., Reinehr T. (2013). Brain-derived neurotrophic factor and its relation to leptin in obese children before and after weight loss. *Metabolism*.

[110] Hohenadel M. G., Thearle M. S., Grice B. A. (2014). Brain-derived neurotrophic factor in human subjects with function-altering melanocortin-4 receptor variants. *International Journal of Obesity (London)*.

[111] Hristova M. G. (2011). Metabolic syndrome and neurotrophins: effects of metformin and non-steroidal antiinflammatory drug treatment. *The Eurasian Journal of Medicine*.

[112] Yoo D. Y., Kim W., Nam S. M. (2011). Reduced cell proliferation and neuroblast differentiation in the dentate gyrus of high fat diet-fed mice are ameliorated by metformin and glimepiride treatment. *Neurochemical Research*.

[113] Patil S. P., Jain P. D., Ghumatkar P. J., Tambe R., Sathaye S. (2014). Neuroprotective effect of metformin in MPTP-induced Parkinson’s disease in mice. *Neuroscience*.

[114] Allard J. S., Perez E. J., Fukui K., Carpenter P., Ingram D. K., de Cabo R. (2016). Prolonged metformin treatment leads to reduced transcription of Nrf2 and neurotrophic factors without cognitive impairment in older C57BL/6J mice. *Behavioural Brain Research*.

[115] Ma J., Liu J., Yu H., Chen Y., Wang Q., Xiang L. (2015). Effect of metformin on Schwann cells under hypoxia condition. *International Journal of Clinical and Experimental Pathology*.

[116] Sola D., Rossi L., Schianca G. P. (2015). Sulfonylureas and their use in clinical practice. *Archives of Medical Science*.

[117] Gong L., Goswami S., Giacomini K. M., Altman R. B., Klein T. E. (2012). Metformin pathways: pharmacokinetics and pharmacodynamics. *Pharmacogenetics and Genomics*.

[118] Rosiak M., Postula M., Kaplon-Cieslicka A. (2013). Metformin treatment may be associated with decreased levels of NT-proBNP in patients with type 2 diabetes. *Advances in Medical Sciences*.

[119] Riphagen I. J., Logtenberg S. J., Groenier K. H. (2015). Is the association of serum sodium with mortality in patients with type 2 diabetes explained by copeptin or NT-proBNP? (ZODIAC-46). *Atherosclerosis*.

[120] Winder W. W., Hardie D. G. (1999). AMP-activated protein kinase, a metabolic master switch: possible roles in type 2 diabetes. *American Journal of Physiology*.

[121] Aune U. L., Ruiz L., Kajimura S. (2013). Isolation and differentiation of stromal vascular cells to beige/brite cells. *Journal of Visualized Experiments*.

[122] Li Y., Wong K., Walsh K., Gao B., Zang M. W. (2013). Retinoic acid receptor beta stimulates hepatic induction of fibroblast growth factor 21 to promote fatty acid oxidation and control whole-body diabetes page 44 of 51 energy homeostasis in mice. *Journal of Biological Chemistry*.

[123] Li Y., Wong K., Giles A. (2014). Hepatic SIRT1 attenuates hepatic steatosis and controls energy balance in mice by inducing fibroblast growth factor 21. *Gastroenterology*.

[124] Wang Q. A., Scherer P. E., Gupta R. K. (2014). Improved methodologies for the study of adipose biology: insights gained and opportunities ahead. *The Journal of Lipid Research*.

[125] Zang M., Xu S., Maitland-Toolan K. A. (2006). Polyphenols stimulate AMP-activated protein kinase, lower lipids, and inhibit accelerated atherosclerosis in diabetic LDL receptor-deficient mice. *Diabetes*.

[126] Stith B. J., Goalstone M. L., Espinoza R., Mossel C., Roberts D., Wiernsperger N. (1996). The antidiabetic drug metformin elevates receptor tyrosine kinase activity and inositol 1,4,5-trisphosphate mass in Xenopus oocytes. *Endocrinology*.

[127] Santos R. F., Nomizo R., Bopsco A., Wajchenberg B. L., Reaven G. M., Azhar S. (1997). Effect of metformin on insulin-stimulated tyrosine kinase activity of erythrocytes from obese women with normal glucose tolerance. *Diabetes & Metabolism*.

[128] Stith B. J., Woronoff K., Wiernsperger N. (1998). Stimulation of the intracellular portion of the human insulin receptor by the antidiabetic drug metformin. *Biochemical Pharmacology*.

[129] Tsai S. J. (2012). Peripheral administration of brain-derived neurotrophic factor to Rett syndrome animal model: a possible approach for the treatment of Rett syndrome. *Medical Science Monitor*.

[130] BDNF Study Group (1999). A controlled trial of recombinant methionyl human BDNF in ALS: the BDNF study group (phase III). *Neurology*.

[131] Géral C., Angelova A., Lesieur S. (2013). From molecular to nanotechnology strategies for delivery of neurotrophins: emphasis on brain-derived neurotrophic factor (BDNF). *Pharmaceutics*.

[132] Kordower J. H., Palfi S., Chen E. Y. (1999). Clinicopathological findings following intraventricular glial-derived neurotrophic factor treatment in a patient with Parkinson’s disease. *Annals of Neurology*.

[133] Li L., Yu T., Yu L., Li H., Liu Y., Wang D. (2016). Exogenous brain-derived neurotrophic factor relieves pain symptoms of diabetic rats by reducing excitability of dorsal root ganglion neurons. *International Journal of Neuroscience*.

[134] Morgado C., Pereira-Terra P., Cruz C. D., Tavares I. (2011). Minocycline completely reverses mechanical hyperalgesia in diabetic rats through microglia-induced changes in the expression of the potassium chloride co-transporter 2 (KCC2) at the spinal cord. *Diabetes, Obesity and Metabolism*.

[135] Seki M., Tanaka T., Nawa H. (2004). Involvement of brain-derived neurotrophic factor in early retinal neuropathy of streptozotocin-induced diabetes in rats: therapeutic potential of brain-derived neurotrophic factor for dopaminergic amacrine cells. *Diabetes*.

[136] Zheng L. R., Zhang Y. Y., Han J. (2015). Nerve growth factor rescues diabetic mice heart after ischemia/reperfusion injury via up-regulation of the TRPV1 receptor. *Journal of Diabetes and its Complications*.

[137] Walwyn W. M., Matsuka Y., Arai D. (2006). HSV-1-mediated NGF delivery delays nociceptive deficits in a genetic model of diabetic neuropathy. *Experimental Neurology*.

[138] Lyons W. E., Mamounas L. A., Ricaurte G. A. (1999). Brain-derived neurotrophic factor-deficient mice develop aggressiveness and hyperphagia in conjunction with brain serotonergic abnormalities. *Proceedings of the National Academy of Sciences of the United States of America*.

[139] Kernie S. G., Liebl D. J., Parada L. F. (2000). BDNF regulates eating behavior and locomotor activity in mice. *The EMBO Journal*.

[140] Rios M., Fan G., Fekete C. (2001). Conditional deletion of brain-derived neurotrophic factor in the postnatal brain leads to obesity and hyperactivity. *Molecular Endocrinology*.

[141] Yamanaka M., Itakura Y., Tsuchida A., Nakagawa T., Noguchi H., Taiji M. (2007). Comparison of the antidiabetic effects of brain-derived neurotrophic factor and thiazolidinediones in obese diabetic mice. *Diabetes, Obesity and Metabolism*.

[142] Bretzner F., Liu J., Currie E., Roskams A. J., Tetzlaff W. (2008). Undesired effects of a combinatorial treatment for spinal cord injury--transplantation of olfactory ensheathing cells and BDNF infusion to the red nucleus. *European Journal of Neuroscience*.

[143] Pearse R. N., Swendeman S. L., Li Y., Rafii D., Hempstead B. L. (2005). A neurotrophin axis in myeloma: TrkB and BDNF promote tumor-cell survival. *Blood*.

[144] Vanhecke E., Adriaenssens E., Verbeke S. (2011). Brain-derived neurotrophic factor and neurotrophin-4/5 are expressed in breast cancer and can be targeted to inhibit tumor cell survival. *Clinical Cancer Research*.

